# Generalized sleep decoding with basal ganglia signals in multiple movement disorders

**DOI:** 10.1038/s41746-024-01115-7

**Published:** 2024-05-10

**Authors:** Zixiao Yin, Huiling Yu, Tianshuo Yuan, Clay Smyth, Md Fahim Anjum, Guanyu Zhu, Ruoyu Ma, Yichen Xu, Qi An, Yifei Gan, Timon Merk, Guofan Qin, Hutao Xie, Ning Zhang, Chunxue Wang, Yin Jiang, Fangang Meng, Anchao Yang, Wolf-Julian Neumann, Philip Starr, Simon Little, Luming Li, Jianguo Zhang

**Affiliations:** 1 Department of Neurosurgery, Beijing Tiantan Hospital, Capital Medical University, Beijing, China; 2 Movement Disorder and Neuromodulation Unit, Department of Neurology, Charité—Campus Mitte, Charite—Universitatsmedizin Berlin, Chariteplatz 1, 10117 Berlin, Germany; 3 National Engineering Research Center of Neuromodulation, School of Aerospace Engineering, Tsinghua University, 100084 Beijing, China; 4 Department of Bioengineering, University of California, San Francisco, UCSF Byers Hall Box 2520, 1700 Fourth St Ste 203, San Francisco, CA 94143 USA; 5 Department of Neurology, University of California, San Francisco, 1651 4th Street, San Francisco, CA 94158 USA; 6 Department of Neuropsychiatry, Behavioral Neurology and Sleep Center, Beijing Tiantan Hospital, Capital Medical University, Beijing, China; 7 Department of Functional Neurosurgery, Beijing Neurosurgical Institute, Capital Medical University, Beijing, China; 8 Department of Neurosurgery, University of California, San Francisco, Eighth Floor, 400 Parnassus Ave, San Francisco, CA 94143 USA; 9 IDG/McGovern Institute for Brain Research, Tsinghua University, 100084 Beijing, China; 10 Beijing Key Laboratory of Neurostimulation, Beijing, China

**Keywords:** Sleep disorders, Movement disorders, Biomedical engineering

## Abstract

Sleep disturbances profoundly affect the quality of life in individuals with neurological disorders. Closed-loop deep brain stimulation (DBS) holds promise for alleviating sleep symptoms, however, this technique necessitates automated sleep stage decoding from intracranial signals. We leveraged overnight data from 121 patients with movement disorders (Parkinson’s disease, Essential Tremor, Dystonia, Essential Tremor, Huntington’s disease, and Tourette’s syndrome) in whom synchronized polysomnograms and basal ganglia local field potentials were recorded, to develop a generalized, multi-class, sleep specific decoder – *BGOOSE*. This generalized model achieved 85% average accuracy across patients and across disease conditions, even in the presence of recordings from different basal ganglia targets. Furthermore, we also investigated the role of electrocorticography on decoding performances and proposed an optimal decoding map, which was shown to facilitate channel selection for optimal model performances. *BGOOSE* emerges as a powerful tool for generalized sleep decoding, offering exciting potentials for the precision stimulation delivery of DBS and better management of sleep disturbances in movement disorders.

## Introduction

A considerable fraction of the world’s population is affected by sleep disorders, which result in substantial welfare expenses^1–3^. Irregular sleep patterns and related disorders are robust indicators of morbidity and mortality across all causes^4,5^. Specifically, movement disorders patients are a population strongly affected by sleep disorders^6^, which profoundly impact their quality of life and potentially hasten the disease progression^7^. Deep brain stimulation (DBS) is a therapy widely used across multiple movement disorders, effectively ameliorating motor symptoms while also contributing to the improvement of sleep disturbances^8,9^. Importantly, the recent advent of the adaptive closed-loop DBS^10^ offers the unprecedented promise of enhancing sleep quality further through sleep stage-specific stimulation^11,12^. However, this advancement poses the need for automatic decoding of a patient’s sleep-wake cycle.

Typically, the classification of sleep stages necessitates intricate laboratory-level polysomnography (PSG) monitoring. Although wearable devices like actigraphy^13^ and photoplethysmography^14^ have the potential for sleep staging, their data is lower dimensional than neural signals, resulting in limited classification accuracies^15^. Actigraphy-based classification in populations with movement disorders will likely also be challenging and require formal validation. Moreover, dependency on additional wearable devices will reduce utility, especially in elderly populations.

Prior research, including our own^16–19^, has attempted to decode sleep stages based on basal ganglia local field potentials (LFPs) recorded from DBS electrodes. This approach has proven to be feasible and potentially reduces the use case for additional wearable devices. However, existing research has predominantly focused on individualized models, where training and prediction are conducted using data from the same patient. This approach requires sleep labeling for each new patient. In clinical practice, the acquisition of laboratory-level sleep recordings for every DBS patient to establish individualized sleep staging models would not be tractable or scaleable^20^. In this study, we addressed this challenge by establishing the largest to date synchronized basal ganglia LFP - PSG dataset in a cohort of 121 patients with movement disorders. With this dataset, we trained a sleep decoding model, termed *BGOOSE* (colloquially “big goose”), the Basal Ganglia Oscillation-based Model for Sleep Stage Estimation, that could decode sleep stages across individuals, disease entities, and even basal ganglia nucleus targets. We also investigated the role of additional electrocorticography (ECoG) in sleep decoding and established the projection between electrode localization and sleep decoding accuracy. Finally, we validated our classification model on two external datasets recorded at different postoperative time points and using different DBS devices.

## Results

### Patient demographics and determination of the best decoder

**Fig. 1:**
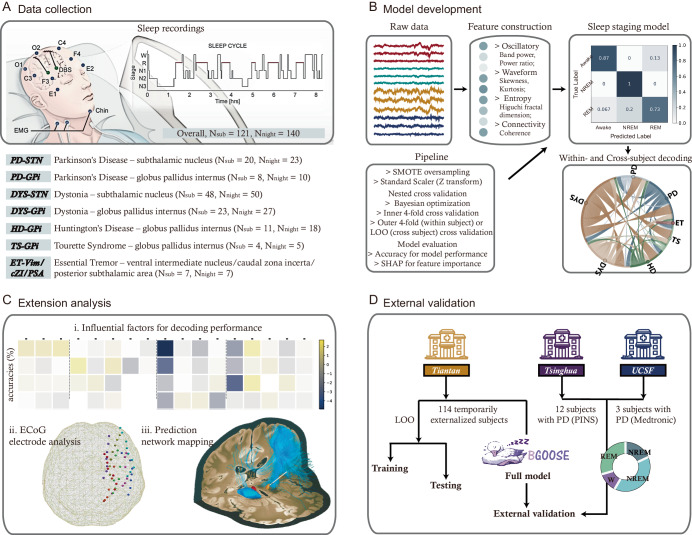
The training and prediction pipeline of the *BGOOSE*. *BGOOSE* is the acronym for the basal ganglia oscillation-based model for sleep stage estimation. **A** Diagram for synchronized basal ganglia and polysomnography recordings in a cohort of 121 patients who underwent DBS surgery (adapted from Yin et al.^32^). **B** Pipeline for model development. See “Methods” for more details. **C** The extension analysis includes: (i) moderator analysis investigating factors that may influence decoding accuracies; (ii) evaluating model performance after taking ECoG signals into consideration; and (iii) prediction networking mapping analysis which aims at establishing the projection between channel localization and sleep decoding accuracy (adapted from Merk et al.^27^). **D** The performance of *BGOOSE* is validated in two external datasets where basal ganglia local field potentials were recorded using sensing-enable devices during sleep.

We recorded 169 overnight (80,430.5 min in bed) synchronized polysomnogram and basal ganglia field potentials in 141 patients with movement disorders who were treated with DBS. Sleep stage was determined both manually and algorithmically^21^ based on standard rules^22^ every 30 s and only epochs with consistent judgments between human experts and algorithms were qualified for further analyses (on average *n* epochs = 621.9 per night). After the exclusion of recordings with a low count of NREM or REM sleep (< 5 min), 140 overnight recordings from 121 patients including Parkinson’s disease-subthalamic nucleus (PD-STN, *n* = 20), PD-globus pallidus internus (PD-GPi, *n* = 8), Dystonia-STN (*n* = 48), Dystonia-GPi (*n* = 23), Huntington’s disease (HD) -GPi (*n* = 11), Tourette syndrome (TS)-GPi (*n* = 4) and Essential tremor-ventral intermediate nucleus/caudal zona incerta/posterior subthalamic area (ET-vim/CZi/PSA, *n* = 7) were analyzed (Fig. 1A). Demographics, disease information, and sleep parameters of each disease-target group are shown in Table 1. Previous sleep decoding studies employing different classifiers have obtained varied accuracies^16–19^. We evaluated the performance of eight commonly used machine learning classifiers in our PD-STN dataset. It is shown that the LightGBM classifier, a lightweight gradient boosting framework based on decision tree algorithm^23,24^ that has been used in multiple sleep-related studies^21,25^, was constantly associated with the highest accuracies as well as a sensible model convergent speed in both the three-class (wake/NREM/REM) and five-class (wake/N1/N2/N3/REM) decoding contexts (Supplementary Fig. 1). We therefore based our subsequent analysis on the LightGBM classifier.

**Table 1:** Patient demographics and sleep parameters

	All-subjects (*n* = 121)	PD-STN (*n* = 20)	PD-GPi (*n* = 8)	DYS-STN (*n* = 48)	DYS-GPi (*n* = 23)	HD-GPi (*n* = 11)	TS-GPi (*n* = 4)	ET^a^ (*n* = 7)
Age	49.7 ± 16.9	62.1 ± 10.7	51.9 ± 21.0	47.7 ± 16.8	47.1 ± 16.2	47.1 ± 11.1	20.8 ± 2.2	56.1 ± 16.2
Sex (male)	49 (40.5%)	8 (40.0%)	6 (75.0%)	18 (37.5%)	9 (39.1%)	6 (54.5%)	1 (25.0%)	1 (14.3%)
BMI	22.4 ± 3.3	24.0 ± 3.4	21.4 ± 2.9	22.3 ± 3.5	22.0 ± 2.5	21.5 ± 1.5	24.9 ± 7.3	20.2 ± 2.5
Disease duration (year)	–	9.8 ± 5.7	8.6 ± 5.6	7.1 ± 8.3	2.8 ± 1.9	6.1 ± 4.0	12.2 ± 3.9	12.0 ± 11.6
Disease severity^b^	–	40.2 ± 12.9	43.9 ± 18.1	21.3 ± 23.1	14.7 ± 11.8	63.2 ± 12.4	71.2 ± 5.6	36.1 ± 5.9
Sleep structure (%)^c^	9.8/62.2/10.1/17.8	11.3/62.3/9.6/16.8	7.9/62.8/12.9/16.4	10.9/60.2/11.3/17.6	8.5/63.5/8.3/19.7	8.3/68.7/5.9/17.1	3.9/59.0/13.2/23.9	11.2/62.6/11.0/15.2
Sleep latency (min)^d^	25.2 ± 31.2	38.2 ± 42.9	30.1 ± 37.9	26.6 ± 32.3	21.1 ± 21.6	14.5 ± 22.5	16.4 ± 11.5	9.7 ± 7.7
Sleep efficiency (%)^e^	72.8 ± 14.6	70.3 ± 11.4	73.0 ± 16.5	73.3 ± 15.6	75.4 ± 12.8	69.8 ± 13.2	83.6 ± 6.5	65.8 ± 21.6
Sleep segmentation (n)^f^	12.6 ± 6.8	12.9 ± 5.5	11.2 ± 4.5	12.7 ± 7.6	10.0 ± 5.6	18.5 ± 7.8	10.2 ± 5.3	14.0 ± 6.2

*PD* Parkinson’s disease, *STN* subthalamic nucleus, *GPi* globus pallidus internus, *DYS* dystonia, *HD* Huntington’s disease, *TS* Tourette syndrome, *ET* essential tremor, *BMI* body mass index.

^a^Targets for ET included ventralis intermediate nucleus, caudal zona incerta, subthalamic nucleus, and posterior subthalamic area.

^b^The presented disease severity scores were the MDS-Unified Parkinson’s Disease Rating Scale section III off-medication score for Parkinson’s disease, the Burke–Fahn–Marsden Dystonia Rating Scale-Movement score for dystonia, the Unified Huntington’s Disease Rating Scale score for Huntington’s disease, the Yale Global Tic Severity Scale total score for Tourette syndrome, and the Essential Tremor Rating Assessment Scale score for essential tremor.

^c^Data presented represent the percentage of N1/N2/N3/REM sleep.

^d^Sleep latency is defined as the time from lights out until the first epoch of any stage of sleep.

^e^Sleep efficiency is defined as the ratio of total sleep time to time in bed.

^f^Sleep segmentation is defined as the times that sleep is interrupted by > 2 min of wakefulness.

### Individualized sleep decoding in different movement disorders

**Fig. 2:**
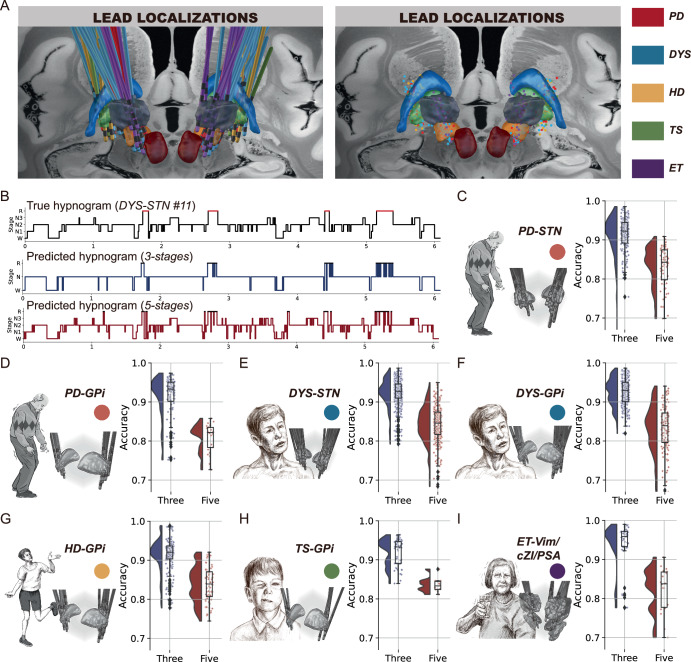
Individualized sleep decoding with basal ganglia signals for patients with movement disorders. **A** shows lead (left) and contact (right) localizations for all subjects. **B** shows the true and predicted hypnograms from a representative patient (*DYS-STN #11*). **C–I** shows the lead localization and results of individualized sleep decoding (training and testing the model using data from the same subject) in each movement disorders cohort. The raincloud plot shows a cloud of individual raw data points, a box plot, and a one-sided violin plot. For the boxplot, the lower and upper borders of the box represent the 25th and 75th percentiles, respectively. The centerline represents the median. The whiskers extend to the smallest and largest data points that are not outliers (1.5 times the interquartile range). The violin plot shows the probability density of the accuracies at different values. The portrait of the patient with Parkinson’s disease is adapted from Arora et al.^66^.

We then built individualized models to investigate whether sleep decoding with basal ganglia signals was feasible in other disease entities than PD and other basal ganglia nuclei than the STN. All subcortical recording sites are shown in Fig. 2A. Results showed that field potentials recorded from multiple basal ganglia nuclei including the STN, GPi, Vim, CZi, and PSA all enabled satisfactory classifications of sleep-wake state in multiple disorders including PD, DYS, HD and ET (Fig. 2B–I). The average accuracy was 94.1 ± 0.8% (range 93.7–95.7%) for the three-class classification and 86.5 ± 1.1% (range 84.7–87.6%) for the five-class classification across all groups.

### Cross-subject, cross-disease, cross-target, and across-all decoding

**Fig. 3:**
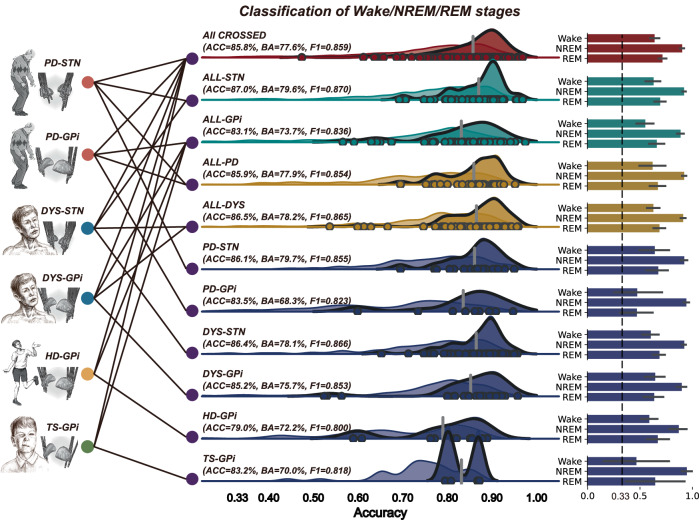
Cross-subject sleep decoding with basal ganglia signals for patients with movement disorders. Eleven decoding contexts are generated from different combinations of 7 movement disorder cohorts. For the *ALL-CROSSED* condition, all subject’s data (*n* = 114) were used for leave-one-subject-out cross-validation (LOOCV) regardless of the disease and target differences. For the *ALL-STN* and *ALL-GPi* conditions, data from patients who had implantations in the STN (*n* = 67) and GPi (*n* = 47) were used for LOOCV, respectively, regardless of the disease information. For the *ALL-PD* and *ALL-DYS* conditions, data from patients who were diagnosed as PD (*n* = 28) and dystonia (*n* = 71) were used for LOOCV, respectively, regardless of the DBS target information. For the *PD-STN* (*n* = 19), *PD-GPi* (*n* = 9), *DYS-STN* (*n* = 48), *DYS-GPi* (*n* = 23), *HD-GPi* (*n* = 11), and *TS-GPi* (*n* = 4) conditions, data from each disease-target group were used for LOOCV in their individual groups. Decoding accuracies are shown in the middle column, with accuracy (ACC), balanced accuracy (BA), and F1 score showing below the name of the cohort. Each dot represents the accuracy value obtained from the left-out subject in that group. The deep-colored regions in the one-sided violin plots show the probability density of the decoding accuracies obtained from the best decoding channels. The light-colored regions show the decoding accuracies obtained from all channels. The vertical gray line represents the mean accuracy value of the best decoding channels. Decoding accuracies for each stage of wakefulness, NREM, and REM sleep from the best decoding channels are shown in the right column. The dashed black line represents the chance accuracy of 33%. The portrait of the patient with Parkinson’s disease is adapted from Arora et al.^66^.

**Fig. 4:**
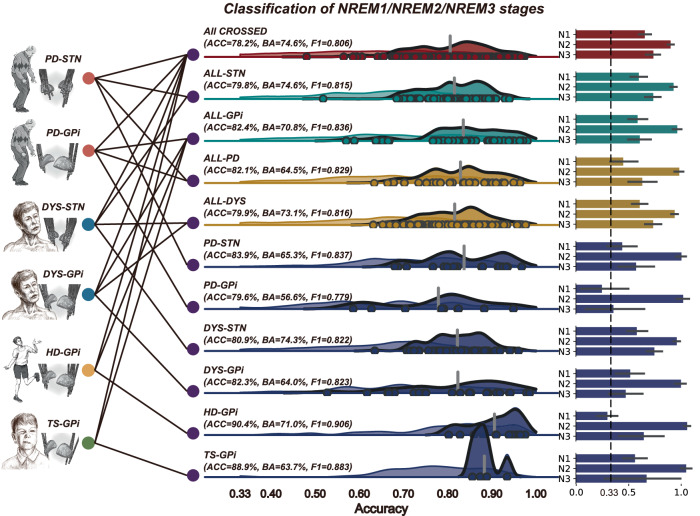
Cross-subject decoding for the classification of NREN 1/2/3 stages with basal ganglia signals for patients with movement disorders. The same convention as in Fig. 3.

Though individualized models demonstrated promising performances, they require cumbersome PSG-based stage labeling and subcortical LFP recordings for model training for each new patient, which could be challenging to obtain in the clinic. We explored whether sleep decoding could be performed in a generalized manner, e.g., training a plug-and-play model that could predict sleep stages in unseen patients. ET group was not analyzed here due to their high heterogeneity in nucleus implantation (e.g., vim/CZi/PSA). Data from the remaining 114 patients were analyzed. In the main analysis, we focused on the three-stage classification of wake/NREM/REM while a further attempt to classify NREM substages (i.e., NREM 1/2/3) was performed as a side analysis. Through a rigorous leave-one-subject-out cross-validation approach (the number of sleep stages used for training and testing is provided in Supplementary Table 1), results (Fig. 3) showed that in the same-disease, same-target, cross-subject manner, the average accuracy of the generalized decoders was 83.9 ± 2.7%. For the same-disease, cross-target decoding, the average generalized decoding accuracy was 85.1 ± 2.8%. For the cross-disease, same-target decoding, the average generalized decoding accuracy was 86.2 ± 0.5%. When not considering variabilities from disease, target, and subject, the final average accuracy of the “*cross-all*” models remained to be 85.9%. The average mislabeling rate for the *cross-all* model was 8.85 ± 5.1 min per hour (Supplementary Table 2). For each stage, the accuracies of the *cross-all* model in classifying wakefulness, NREM, and REM sleep were 64.2%, 90.6%, and 71.8% respectively, with an overall balanced accuracy of 77.6% (Supplementary Fig. 2) and a weighted F1 score of 0.859 (Supplementary Fig. 3). Feature importance analysis with SHAP (SHapley Additive exPlanations) showed that delta/theta power ratio was the most contributed feature for the *cross-all* decoding following by theta power and permutation entropy (Supplementary Fig. 4). For the further classification of NREM 1/2/3 stages, the *cross-all* model obtained an average accuracy of 78.2%, a balanced accuracy of 74.6% and a weighted F1 score of 0.806, with the identification of NREM2 sleep showing the highest average accuracy of 80.2% (Fig. 4).

### Additional electrocorticography helps sleep decoding

**Fig. 5:**
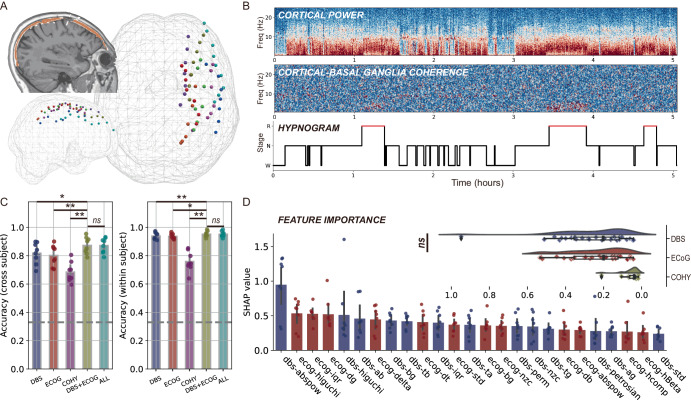
Additional electrocorticography (ECoG) electrode improves sleep decoding. **A** shows the localization of all temporary ECoG electrodes in a glass brain. **B** shows an example time-frequency representation of cortical power (upper), cortical-basal ganglia coherence (middle), and the corresponding hypnogram (bottom) in a representative subject. **C** shows the decoding accuracies using DBS channel features, ECoG channel features, cortical-basal ganglia coherence features (COHY), DBS plus ECoG channel features, and all features together (ALL) in the contexts of within-subject decoding (left) and cross-subject decoding (right). The gray dashed horizontal lines indicate a chance accuracy of 33%. * *P* < 0.05, ** *P* < 0.01. **D** shows the feature importance when conducting cross-subject decoding with all features. ECoG features are as important as basal ganglia features (*P* = 0.993, Mann–Whitney *U* test), though both features are more important than coherence features, as shown in the inset. ns, non-significant.

Accumulating evidence shows that electrocorticography (ECoG) adds accuracy in informing state-dependent adaptive DBS^26–28^. Here we investigated whether ECoG signals could outperform basal ganglia ones in sleep decoding and whether a combination of both signals adds benefit to each alone. In 8 temporarily externalized subjects (6 PD, 2 DYS), 11 overnight ECoG data were obtained together with basal ganglia and PSG recordings. A total of 58 ECoG channels were analyzed (shown in Fig. 5A). We extracted ECoG features in frequency, time, and entropy domain following the same manner as subcortical signals. Cortical-subcortical connectivity was quantified as coherence (exemplified in Fig. 5B). Results showed that cortical and subcortical features resulted in comparable sleep decoding accuracies, both significantly higher than that obtained by connectivity features (Fig. 5C). A combination of subcortical and cortical features significantly enhanced decoding accuracy compared to each feature alone, especially for the cross-subject decoding (Fig. 5C), with no further accuracy improvement after adding connectivity features (Fig. 5C). Subcortical and cortical features were equally important based on SHAP analysis (Fig. 5D).

### Moderator analysis and prediction of best decoding channels

**Fig. 6:**
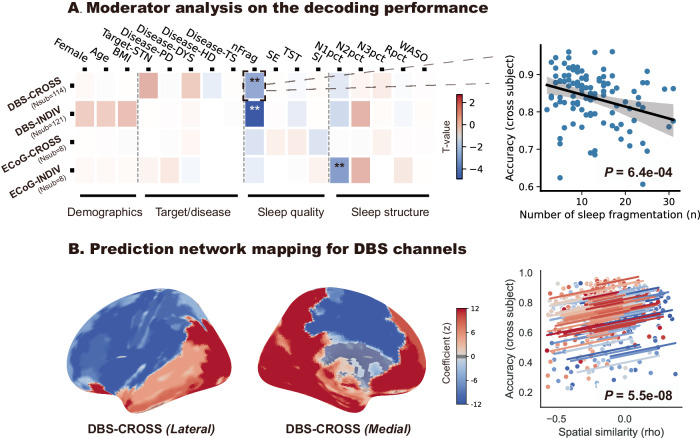
Moderator analysis and network mapping of the decoding accuracies. **A** Heatmap showing the influential factors of decoding accuracies in different contexts including within-subject decoding with basal ganglia data (DBS-INDIV), cross-subject decoding with basal ganglia data (DBS-CROSS), within-subject decoding with ECoG data (ECoG-INDIV), and cross-subject decoding with ECoG data (ECoG-CROSS). Candidate factors are elaborated on in the Methods section. The right side shows the regression plot which depicts the correlation between the number of sleep fragmentations and the decoding performances. Significant associations with *p* values < 0.0029 (0.05/17) were highlighted with asterisks. **B** shows the lateral and medial view of the optimal decoding map for the basal ganglia electrodes. The map was generated by first generating a volume of tissue recorded with a radius of 5 mm for each contact and then calculating the connectivity pattern between the seed volume and the normative functional MRI connectome. The obtained whole-brain connectivity strength was then voxel-wised correlated with the decoding accuracy, resulting in the final optimal decoding map. Increased projection to the purple area indicates a higher chance to obtain good decoding results while increased projection to the blue area indicates a lower chance to obtain good decoding results. The right panel shows the repeated measurement regression plot between the spatial similarity to the optimal map and the decoding accuracies obtained in a leave-one-subject-out manner.

We next investigated what factors may influence sleep decoding accuracies. Linear mixed-effect models showed that patient demographics and disease/target information had no significant impact on decoding performances in either individualized or generalized decodings using basal ganglia and cortical features (Fig. 6A, left). Poorer sleep quality as assessed by a higher count of sleep fragmentation was significantly associated with lower decoding accuracies in basal ganglia-based models (individualized model: coef = −0.00189, *p* = 1.01e–6; generalized model: coef = −0.00317, *p* = 6.39e-4, Fig. 6A, right). A higher proportion of N1 sleep was also associated with lower decoding accuracies in cortical-based individualized models (coef = −0.00265, *p* = 2.90e-4). We also evaluated the effects of various re-referencing methods on the decoding performance of DBS channels. Specifically, we compared the adjacent referencing method (e.g., the 1–2 re-referenced channel for the 1-2-3-4 contact DBS lead) and the ‘sandwich’ referencing method (e.g., the 1–3 re-referenced channel). We found that the sandwich montage channels showed significantly higher average decoding accuracies than the adjacent re-referenced channels in both the generalized and individualized models (Supplementary Fig. 5), a finding that could be relevant to the application of adaptive DBS.

As previous works showed^18,27^, the anatomical location of the recording electrode was also found to have an impact on decoding performance. This relationship is important since for an unseen subject we are required to determine the best possible decoding channel without having prior knowledge. Within the framework of prediction network mapping^27^, we simulated a volume of tissue recorded^18^ for each basal ganglia LFP channel and calculated its connectivity with normative functional MRI connectome^29^. We then correlated the connectivity strength with decoding accuracy to establish the projection between channel localization and decoding performance. This generated a whole-brain optimal decoding map (Fig. 6B, left). Increased projections to the temporal lobe were associated with higher decoding performances while increased projections to the parietal lobe were associated with lower decoding accuracies, across all basal ganglia seed targets. We validated this prediction network mapping approach by constructing a map for all patients minus one and then using the constructed map to predict the left-out unseen patients, repeating until all patients were predicted. Results suggested that in this leave-one-subject-out manner, higher spatial similarity (i.e., Spearman rho) between the basal ganglia channel’s projection map and the optimal map constructed using all-minus-one data significantly predicted higher decoding accuracies (coef = 0.233, *P* = 5.50e-8, linear mixed effect model, Fig. 6B, right), supporting the utility of the optimal map in predicting the best decoding channels in unseen subjects based solely on channel localizations. Note that only adjacent but not sandwich re-referenced channels were employed here as sandwich channels may have different sizes of volume recorded and signal-to-noise ratios. We also attempted to construct an optimal decoding map for ECoG channels. However, for ECoG this approach did not yield significant predictions of decoding accuracy (Supplementary Fig. 6).

### External validations using chronically embedded sensing-enabled devices

**Fig. 7:**
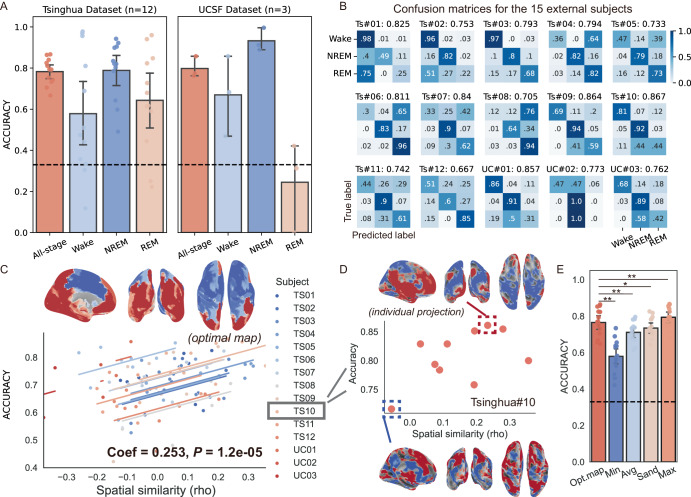
External validation for the *BGOOSE.* **A** shows the average accuracy for the *BGOOSE* to classify sleep stages in two external cohorts with basal ganglia recordings during sleep. The lower and upper borders of the box represent the 25th and 75th percentiles, respectively. The centerline represents the median. The whiskers extend to the smallest and largest data points that are not outliers (1.5 times the interquartile range). The black dashed line represents the chance accuracy of 33%. **B** shows the decoding confusion matrices for each of the 15 unseen subjects in the two cohorts. **C** demonstrates the repeated measurement correlation between spatial similarity to the optimal map and the decoding accuracy. The upper inset shows the medial, posterior, and dorsal views of the optimal decoding map. **D** shows the correlation between spatial similarity and decoding accuracy in one representative patient (Ts#10). The channel with higher decoding accuracy (upper inset) has higher whole-brain projection similarity to the optimal map than the channel with lower decoding accuracy (bottom inset). **E** shows the comparison between mean accuracy values obtained from optimal-map selected channels (Opt.map), channels with worst decoding performances (Min), all channels (Avg), “sandwich” referenced channels (Sand), and channels with best decoding performances (Max). Same conventions as in Fig. 7A. *P* = 9.82×10^−4^ for the comparison of accuracy between map-based channels and worst decoding channels. *P* = 2.01×10^−3^ for the comparison of accuracy between map-based channels and all channels. *P* = 3.02×10^−2^ for the comparison of accuracy between map-based channels and sandwich re-referenced channels. *P* = 7.69×10^−3^ for the comparison of accuracy between map-based channels and best decoding channels. Wilcoxon signed-rank test.

In the final part, all patients’ data (*n* = 114) were utilized to train a generalized sleep decoding model which we termed *BGOOSE*. Two external datasets, the Tsinghua dataset, which included 12 PD patients with STN LFPs recorded using the PINS sensing-enabled devices, and the UCSF dataset, which included 3 PD patients with basal ganglia LFPs (2 STN, 1 GPi) recorded using the Medtronic sensing-enabled RC + S Summit devices were used to test the performance of *BGOOSE*. Both datasets were obtained at least one month after electrode implantation. Patient information is shown in Supplementary Table 3. The average decoding accuracies were 78.3 ± 6.1% and 79.8 ± 4.2% for the Tsinghua and UCSF datasets, respectively, ranging between 86.7% and 66.7% for each individual (Fig. 7A, B). For different stages, average accuracy was the highest for NREM sleep: 81.7%, and was the lowest for REM stage: 56.3%, with an overall mislabeling rate of 12.8 ± 3.4 min per hour. A further subdivision of NREM sleep into N1/N2/N3 stages demonstrated high accuracies in identifying N2 sleep (90.7%), while the accuracies of classifying N1 (43.9%) and N3 (5.6%) stages were low (Supplementary Fig. 7). Decoding performances across all 15 subjects in classifying wake/NREM/REM stages were significantly predicted by the optimal decoding map (Fig. 7C). Channels predicted to have good performances had significantly higher accuracies than all-channel average accuracies, though still lower than the theoretically best ones (Fig. 7D, E).

## Discussion

**Fig. 8:**
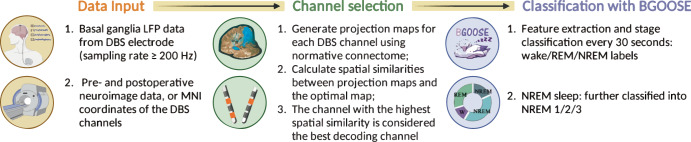
Pipeline for the optimal use of BGOOSE. A pipeline summarizing the main function of *BGOOSE* and how can it be optimally used.

We developed a novel sleep staging tool named *BGOOSE* (colloquially “big goose”), designed for the automated decoding of sleep stages using local field potentials recorded from DBS electrodes in movement disorders. The development of *BGOOSE* was preceded by the establishment of the largest known database of sleep-related basal ganglia electrophysiological recordings, wherein precise sleep stage annotations were applied through both manual and algorithmic methods. Subsequently, our study demonstrated the feasibility of generalized sleep decoding, achieving an average decoding accuracy of approximately 85% in a cross-patient, cross-disease, and cross-basal ganglia structure scenario for the classification of wake/NREM/REM stages. Moreover, we proved that the inclusion of additional intracranial ECoG electrodes in the frontoparietal cortex further enhanced sleep stage decoding accuracy, which might be particularly important in patients with sleep fragmentation who showed lower decoding accuracy. By mapping the relationship between electrode contact locations and decoding accuracy, we provided an approach for channel selection for optimal sleep decoding performance, using neuroimaging data only. Finally, the generalizability of our approach was validated in two independent external validation datasets collected using different DBS devices. A pipeline showing how *BGOOSE* could be optimally used for sleep stage decoding is provided in Fig. 8.

Prior research on automatic sleep staging has predominantly been based on EEG data or SEEG data^30,31^. The demand for sleep state decoding using basal ganglia LFP data has arisen dramatically due to the recent application of adaptive DBS which could potentially better address sleep disturbances through sleep stage-specific stimulation^10,32^. One of the earliest models for automatic sleep staging using basal ganglia signals was proposed by Thompson et al. in^19^, who trained a support vector machines (SVM)-based model with STN LFPs for sleep decoding in 10 patients with PD. The model exhibited promising performance in individualized scenarios, achieving approximately 90% overall accuracy in classifying awake/NREM/REM states, but fell short in cross-subject scenarios with an average accuracy of about 50%. Subsequent studies^16,17^ explored the use of artificial neural network (ANN), a type of deep learning technology, and decision tree models for sleep staging in PD-STN patients, with similar performance for individualized decoding and around 65% accuracy for cross-subject decoding. Our previous work^18^ trained random forest models with over 500 h of pallidal LFP data recorded during sleep but still only obtained a cross-subject decoding accuracy of 65.1%. Given that the patterns of basal ganglia activity across sleep stages were different from patient to patient^19^ and that diseases were likely to further exert alterations on brain activity^18^, it could be challenging to build a generalized model for all movement disorders with acceptable accuracies. Here in this study, the *BGOOSE* model we presented, based on the LightGBM gradient boosting framework^23^, achieved approximately 85% sleep staging accuracy in a generalized context and maintained around 80% accuracy during out-of-cohort validations. Given that the interrater reliability of sleep stage scoring for human experts is around 80%^33^, we considered that the accuracies generated by *BGOOSE* could be good enough to use even though we addressed only the three-stage rather than the five-stage problem. In the exact use case of closed-loop DBS, since there are currently very few, if not no, proposed reasonable acceptance criteria needed for effective closed-loop DBS during sleep, it is difficult to interpret how good such accuracies are. In our previous proof-of-principle study^12^, a classifier model was trained to identify N3 sleep epochs using ECoG signals to inform adaptive DBS during sleep. The decoder achieved high specificity (0.94 ± 1.4e–2) and well above chance sensitivity (0.62 ± 4e–2) with an overall accuracy of around 85%. With this staging performance, the aDBS for sleep stage targeting can already have a significant impact on sleep structures. Based on the mislabeling rate of 9–12 min per hour with *BGOOSE* presented here, it could be inferred that approximately 6 h of sleep can be correctly labeled in a given night of 7-h sleep. This should reasonably provide a remarkable change to the current constant-parameter stimulation strategy during the night.

In the present study, 28 features were constructed for the training of the *BGOOSE* model, including oscillatory features, waveform features, and nonlinear entropy features. These features were selected based on previous work in features-based automatic sleep staging algorithms using EEG or LFP signals^16,18,19,21,34^. For example, it was shown that the permutation entropy of the EEG^21^ and LFP spectral powers^16^ are among the most contributed features for accurate sleep staging. We found that the LFP power ratio between the delta and theta band was the most important feature for cross-subject sleep decoding. This could be because delta and theta oscillations are the defining features of NREM^35^ and REM^36,37^ sleep, respectively. Therefore, it is sensible to speculate that a high, low, and medium delta/theta power ratio may indicate NREM, REM, and awake stages, respectively, although this is not necessarily applicable to all subjects, as in patients with low decoding accuracies the rank of feature importance was very different from that in the whole cohort (Supplementary Fig. 8). Importantly, the features included in the current study were chosen to be computationally efficient for the future online implementation. Other features, which are more computationally intensive and have been demonstrated to differentiate sleep stages including phase-amplitude coupling^38^ and oscillatory bursts^32^ can also be included in future matrices with the advent of more powerful embedded microprocessors.

An interesting finding in the moderator analysis was that higher numbers of sleep fragmentation and a higher proportion of N1 sleep were associated with lower decoding accuracy. This finding is in line with what Vallat et al. found in their automated sleep decoding work with EEG signals, where the percentage of N1 sleep and percentage of stage transitions were the two top predictors of worse accuracy^21^. This relationship could be plausible as the N1 stage is associated with the highest interrater variability even for human experts^33^. However, in practical terms, this might imply that the accuracy of automated scoring could diminish in patients with severe sleep disorders, a population who particularly require the most sleep-stage-specific intervention. Adding further sources of neural activities (e.g., ECoG) would help improve decoding accuracies as shown in Fig. 4.

Another factor that could have an impact on decoding accuracy is the re-reference approach for DBS channels. Traditionally, the four-contact DBS lead was re-referenced adjacently, creating three bipolar channels, which is preferable if one wants to magnify local events that occur near the contacts^39^. The advent of adaptive DBS introduced a “sandwich” montage, which has the advantage that stimulation signals can be passively canceled out through “common mode rejection”^20^. We found that sandwich-referenced channels were associated with significantly higher average decoding accuracy than the adjacent referenced channels even when stimulation was not turned on. We speculated that this could be because the 1–3 or 2–4 referenced approach enables the generated channel to record a larger area of neuronal activity, thus potentially enhancing the signal-to-noise ratio and preventing the cancellation of symmetrically recorded local sources. Given that the external dataset involved channels generated using both reference approaches and the decoding accuracies could still be overall predicted by the optimal projection map, we believe that the montage difference would not remarkably influence the application of the optimal projection map in guiding the selection of better decoding channels.

We would like to highlight several key features of *BGOOSE*. (1) The most distinctive characteristic of *BGOOSE* lies in its plug-and-play, generalized decoding capability across various movement disorders. Unlike previous research that often focused on single basal ganglia LFP signals for a specific movement disorder, *BGOOSE* encompasses five of the most common movement disorders treated with DBS, including PD, DYS, HD, TS, and ET, as well as the two most frequently used DBS targets, STN, and GPi^40–42^. This breadth of applicability represents a significant clinical potential with immediate relevance. (2) *BGOOSE* also stands out for its use of single-channel data and exceptionally fast feature extraction and prediction speed. In our testing, *BGOOSE* was able to construct features and perform classification for a 30-s sleep epoch in less than 0.005 s on an Intel i5 consumer laptop. Although it must be noted that hyper-threaded processors are not easily comparable to the single-threaded reduced instruction set processor which aims to conserve size and power as would be found on an implantable pulse generator (IPG), *BGOOSE*’s feature extraction process that avoids computational complex indices is still advantageous for the future implementation of low-latency online decoder within the IPG. Moreover, the advantage of requiring only a single channel’s data is particularly beneficial in the context of aDBS, as stimulation channels typically lack recording functionality^20,43^. (3) *BGOOSE* also explores the integration of ECoG signals, demonstrating the potential for enhanced decoding accuracy when combined with cortical inputs. The chronically implanted ECoG electrodes have been showing increasingly promising potential in assisting state decoding for aDBS^10,12,27,28^. While the current version of *BGOOSE* focuses on generalized models trained with basal ganglia signals due to limited ECoG data, as more ECoG electrodes are implanted in patients with movement disorders, future iterations of *BGOOSE* are expected to incorporate cortical features, further improving decoding performance. (4) Lastly, *BGOOSE*’s provision of an optimal decoding map allows for the identification of potential optimal basal ganglia decoding channels with only lead localization information^18,27^. This feature becomes essential, especially when dealing with new patients where ground truth sleep labels are not available.

*BGOOSE* presents potential applications across several contexts. Firstly, and most directly, it serves as a tool in guiding stage-specific stimulation for closed-loop DBS systems^12^. Current closed-loop algorithms for PD were predominantly informed by wakefulness beta power to adjust stimulation parameters, but beta activity has been shown to vary significantly across different sleep stages^18,44^. Failure to identify a patient’s current sleep state and persistently using the wakeful beta activity level as a threshold may result in either insufficient or excessive stimulation during sleep^32^. With *BGOOSE*, beta thresholds used for informing stimulation can be adjusted in pace with sleep stage transitions (e.g., adjusting the beta threshold to a lower level during NREM sleep so that pathological beta in PD could be better suppressed through stimulation^32,45^). In addition, obtaining the patient’s sleep stage enables the possibility of enhancing specific beneficial waveforms relevant to different sleep stages, such as spindle and slow waves in NREM sleep^46^ and sawtooth waves in REM sleep^47^. Secondly, for patients whose symptoms are not present at night (e.g., essential tremor and Tourette syndrome), being able to obtain sleep/awake classifications based on LFP signals through *BGOOSE* means that we could switch off stimulation automatically during sleep when stimulation is not needed. The concept of “deep brain stimulation holidays” in essential tremor^48^ has long been proposed, where stimulation is temporarily stopped during the night to prevent tolerance to DBS. Given that the adherence of manually switching on & off stimulation might be difficult for patients^49^, *BGOOSE* emerges as a useful tool to realize this aim algorithmically. Thirdly, the application of *BGOOSE* can facilitate the sleep management in patients with movement disorders. Using the DBS device for sleep staging potentially eliminates the need for additional wearable devices, offering valuable 24/7 longitudinal data for drug adjustments and DBS programming^11^. Fourthly, the use of *BGOOSE* has the potential to advance sleep research in movement disorder patients as one of the current barriers of conducting sleep research lies in the cumbersome determination of sleep stages, typically requiring complex PSG monitoring at sleep laboratories^50^. While *BGOOSE* is not presented as a PSG replacement, it can be viewed as an alternative in scenarios where conducting PSG monitoring is challenging due to equipment or environmental constraints, or when patient cooperation is limited. *BGOOSE*, based on basal ganglia signals, can provide sleep labels in these situations, thereby advancing sleep research in patients with movement disorders.

Despite its many advantages, *BGOOSE* has several limitations that warrant future improvement. First, in cross-decoding, while it achieved an accuracy of over 90% for NREM sleep stages, the classification accuracies for the wake and REM stages were approximately 60–70%. This could be because, compared to the more homogenous NREM sleep, the variability among patients in wake and REM stages was larger^32,51^. This can also be supported by the feature importance analysis shown in Supplementary Fig. 8, where patients with low accuracies in cross-subject decoding had very different top-contributed features from the whole cohort (Supplementary Fig. 4). However, it was essential to note that our previous research had suggested that pathological oscillations in NREM sleep were highly relevant to sleep disorders in PD, making this a critical sleep stage to target for higher accuracy decoding^32^. Additionally, as NREM sleep constitutes over 70% of total sleep time, achieving robust identification of NREM sleep remains critical for sleep decoding models. Second, it is worth noting that REM classification was notably poor in the second external dataset (UCSF) in patients recorded chronically using a fully embedded sensing-enabled DBS (Summit RC + S) pacemaker. Differences in e.g., line noise frequency, or frequency-specific normalization effects may make the challenging REM versus NREM distinction difficult to generalize across recording systems. Third, in the generalized scenario, we put our focus on the three-class decoding problem. Although we tried to build decoders to further classify NREM sleep into N1/N2/N3 stages as a side analysis, the performance was not that satisfactory, especially for the N3 stage (Supplementary Fig. 7). One potential reason could be that the reduced proportion of N3 sleep due to sleep disturbances in a considerable portion of movement disorder patients complicates training and generalization. Accumulating more deep sleep data from chronic recordings for model training^45^ or identifying N3 sleep using ECoG electrodes^12^ are potential solutions. Fourth, *BGOOSE* relies on feature extraction and tree-based algorithms for sleep staging recognition, whereas deep learning algorithms have shown significant potential in the sleep classification field^31,52,53^. Although they may require longer training and parameter tuning time, exploring state-of-the-art deep learning algorithms is a worthwhile endeavor. Fifth, since *BGOOSE* is trained using data recorded during externalized periods, it may lack resistance to electrocardiogram (ECG) artifacts. ECG artifacts are common in perceptive data^54^ and have been observed in the Tsinghua and USCF datasets. In addition, the “micro-lesion effect” during the externalized period could also negatively influence the generalized ability of *BGOOSE* as this effect could temporarily change the oscillatory pattern in basal ganglia (e.g., beta power in Parkinson’s disease)^55,56^. Including more chronically recorded data with all types of environmental artifacts in training will enhance *BGOOSE*’s ability to generalize. Sixth, *BGOOSE* is currently trained in the off-stimulation condition, and its performance during on-stimulation states for sleep staging remains unknown, which is a crucial consideration for continuous monitoring and stimulation^45^. Seventh, *BGOOSE* is trained solely for nighttime sleep, and its applicability for accurate daytime napping sleep staging is uncertain. Lastly, *BGOOSE* serves as a tool for automatic sleep staging but the question of how to implement adaptive stimulation following sleep staging remains a critical, unresolved challenge. For instance, the optimal stimulation approach in N1 to induce deep sleep, in N2/3 to enhance beneficiary waves, and in REM sleep to potentially suppress REM sleep behavior disorder are all areas requiring further investigation. Addressing these questions holds the key to realizing the potential of sleep intervention as a therapeutic tool for managing various disorders.

## Methods

### Patients and surgery

A total of 141 patients with movement disorders scheduled to undergo DBS surgery at Beijing Tiantan Hospital were enrolled in the study after obtaining written informed consent. This study is approved by the IRB of Beijing Tiantan Hospital and performed per the Declaration of Helsinki. Inclusion criteria comprised the following: (1) well-defined disease diagnosis; (2) ability to cooperate with whole-night PSG recordings; and (3) absence of structural lesions observed on MRI scans. Under the guidance of a stereotactic frame system, four-contact DBS electrodes were implanted into the predefined basal ganglia regions as per standard protocols^57^. The accuracy of electrode placement was confirmed through intraoperative electrophysiological recordings, temporary stimulation, and postoperative anatomical computed tomography (CT) scans. In six patients with PD and two patients with dystonia, an additional eight-contact ECoG electrode was implanted into the right motor cortex region through the same burr hole for DBS^26^. Following electrode implantation, patients returned to the ward for sleep recordings, which were typically conducted within 3–7 days post-surgery.

### Sleep recordings and staging

During lead externalization, patients underwent sleep recordings for 1–2 consecutive nights following previous routines^32^. Signal recording was conducted using a JE-212 amplifier (Nihon Kohden, Tokyo, Japan) with a sampling rate of 1000/2000 Hz. All drugs that could potentially influence brain state were stopped throughout the recording session, including anti-parkinsonism and anti-dystonia medications, and sleeping pills. Sleep PSG labels were manually scored every 30 s according to the rules outlined in the AASM manual version 2.6. Four sleep parameters were measured and reported in Table 1: the sleep structure, defined as the percentage of N1/N2/N3/REM sleep; the sleep latency, defined as the time from lights out until the first epoch of any stage of sleep; the sleep efficiency, defined as the ratio of total sleep time to time in bed; and the sleep segmentation, defined as the times that sleep is interrupted by over 2 min of wakefulness^32^. In addition, to enhance staging efficiency and reduce the impact of scorer subjectivity on labeling results, this process was aided by an established open-source sleep staging algorithm (https://github.com/raphaelvallat/yasa)^21^, which was trained on over 30,000 h of PSG data. Only sleep epochs with consistent manual and algorithmic judgments were qualified for further sleep decoding analyses. On average, 621.9 sleep epochs per night were included in the analysis. Sleep stages were categorized into either three (awake/NREM/REM) or five categories (awake/N1/N2/N3/REM) in later analysis. Data from each stage needed to be collected for at least 5 min to be included in the analysis.

### ECoG and LFP preprocessing and feature extraction

All signals were Butterworth notch filtered to reject the 50 Hz ambient noise and harmonics, followed by downsampling to 200 Hz. Bipolar re-referencing was applied to all adjacent contacts for ECoG and DBS electrodes. Besides, for DBS electrodes, a “sandwich” re-referencing (e.g., 1–3 and 2–4 referencing for the 1-2-3-4 lead) was also applied to mimic the clinical use case of adaptive DBS^20^. The sandwich-referenced channels were not analyzed in the network mapping section (see below) as they might have different shapes of recording field and signal-to-noise ratios from adjacent-referenced channels. For feature extraction, we computed a total of 28 local features, which included 18 frequency-domain features: one total absolute power, seven relative energy features in different frequency bands (delta 1–4 Hz, theta 4–8 Hz, alpha 8–13 Hz, low beta 13–20 Hz, high beta 20–30 Hz, low gamma 30–45 Hz, high gamma 55–90 Hz), and ten energy ratio features across frequency bands (delta, theta, alpha, beta, gamma *[4* + *3* + *2* + *1]*). Additionally, we computed 10 time-domain features, consisting of seven statistical features, including standard deviation, interquartile range, skewness, kurtosis, number of zero crossings, Hjorth mobility, and Hjorth complexity, and three entropy features, including permutation entropy, Higuchi fractal dimension, and Petrosian fractal dimension^21^. Furthermore, for patients with ECoG implants, in addition to the abovementioned local features, we extracted 18 basal ganglia-cortical connectivity features, comprising coherence in seven frequency bands (delta, theta, alpha, low beta, high beta, low gamma, and high gamma), ten coherence ratio features across five frequency bands (delta, theta, alpha, beta, gamma band *[4* + *3* + *2* + *1]*), and one peak coherence frequency, defined as the frequency at which the coherence peak occurred. Consequently, in scenarios using single-site signals, 28 features were generated, whereas in cases utilizing dual-site signals, 74 features were computed (28*2 + 18).

### Machine learning models

We tested eight machine learning models in our study, namely, ridge regression, SVM, k-nearest neighbors (KNN), decision tree, random forest, XGBoost, LightGBM, and artificial neural network. Ridge regression, SVM, KNN, decision tree, and random forest were implemented using machine learning algorithms available in the scikit-learn library^58^. XGBoost was implemented using the code provided at https://github.com/dmlc/xgboost/tree/master^59^. LightGBM was implemented using the code available at https://github.com/microsoft/LightGBM/tree/master^23^. The artificial neural network was developed based on the TensorFlow framework^60^. Detailed hyperparameter tuning for each model can be found in Supplementary Table 4. Any unspecified parameters were set to their default values.

### Model training, evaluation, and features importance

Model performance was evaluated using nested cross-validation, with both outer and inner cross-validation set to 4 folds. A Bayesian Optimization hyperparameter search, comprising 50 rounds, was employed for hyperparameter tuning using a balanced accuracy as the scoring function. Given the imbalance in the distribution of sleep stage labels, the SMOTE method from the imbalanced-learn library^61^ was utilized to oversample the minority class labels. For the sake of simplicity, model performance was assessed using average accuracy in the main text. Results with balanced accuracy and weighted F1 score were shown when indicated. The speed of model fitting was evaluated as 1/log (fitting time). Feature importance was assessed and compared using the SHAP method, available at https://github.com/shap/shap/tree/master^62^.

### Moderator analysis

In the moderator analysis, we investigated factors that may influence the accuracy of sleep stage decoding. The independent variables encompassed four categories, namely, (1) demographic information, including sex, age, and BMI), (2) disease and target information (e.g., disease: PD/DYS/HD/TS, target: STN/GPi), (3) sleep quality information, including the number of sleep fragmentations, sleep efficiency, total sleep time, and sleep latency, and (4) sleep structure information including the proportion of time spent in each sleep stage. The effects of different referencing approaches on decoding performance were also investigated.

### Localization of DBS and ECoG electrode

We reconstructed the DBS electrodes using the advanced electrode localization pipeline with default settings in Lead-DBS version 2.5.3 (MATLAB 2019b)^63^. ECoG electrodes were reconstructed using the established method with FreeSurfer^64^. The positions of DBS and cortical electrodes were standardized to the MNI template (ICBM 2009b Nonlinear Asymmetric) for group-level analysis.

### Network mapping for choosing the best decoding channel

We implemented a prediction network mapping approach that was previously established^18,27^ to predict potential optimal decoding channels. This method generates a simulated recording field for each recording site (radius = 5 mm) and calculates its functional connectivity with the whole-brain normative connectome (Brain Genomics Superstruct Project-100^29^). By correlating the decoding accuracy of individual channels with the whole-brain connectivity pattern projected from the corresponding recording field, and incorporating significant moderators identified in the moderator analysis as covariates, we generate an optimal projection map featured in the AAL3 atlas with a total number of parcellations of 166^65^. We then calculated the spatial similarity between each channel’s projection map and the optimal projection map (i.e., the Spearman correlation between two 166-length one-dimensional arrays). For an unseen recording channel, the greater the similarity between its functional connectivity pattern with the whole brain and the optimal map, the higher the likelihood it may obtain higher decoding accuracy.

### External validation

We conducted external validation of our model using two separate datasets. The first dataset, referred to as the Tsinghua dataset, comprised 12 PD patients who underwent STN-DBS surgery^16^. The second dataset, the UCSF dataset, included two PD patients who received STN-DBS and one PD patient who received GPi-DBS^45^. All 15 patients underwent overnight synchronous PSG and basal ganglia signal recording in the off-medication & off-stimulation state. Based on the MNI coordinates, no significant targeting differences were found between the validation group and the training cohort. Demographic information of the external subjects can be seen in Supplementary Table 3. More detailed demographics were reported in two original papers: the 12 PD patients in the Tsinghua dataset are the same patients named No.1 to No.12 in Table 1 in Yue Chen et al.^16^, and the 3 PD patients in the UCSF dataset are the same patients named as PD2, PD7, and PD9 in Table 1 in Md Fahim Anjum et al.^45^. The validation process encompassed two aspects: (1) validation of decoding performances of the *BGOOSE* and (2) validation of the prediction of the optimal decoding channel using the optimal projection map.

### Statistical analysis

We aimed to conduct statistical comparisons using non-parametric tests including the Mann–Whitney U test, Wilcoxon signed-rank test, and Spearman correlation wherever possible. We employed linear mixed-effects models for the moderator analysis. The significance threshold for two-sided *P*-values was set at 0.05.

### Reporting summary

Further information on research design is available in the Nature Research Reporting Summary linked to this article.

### Supplementary information

SUPPLEMENTAL MATERIAL

Reporting Summary

## Data Availability

The original data are not yet openly available, as it is being used in ongoing projects. We welcome enquires for sharing this as part of a collaboration, please contact the corresponding authors.

## Code availability

The code for analyzing the data and the final version of the generalized decoding model is made freely available at https://github.com/zixiao-yin/BGOOSE. The sleep decoding models for the three and five-stage classifications can be found at https://osf.io/mt72e/ (10.17605/OSF.IO/MT72E).
