# Supplementary material for: Generalized sleep decoding with basal ganglia signals in multiple movement disorders
Source: NPJ Digit Med. 2024 May 10;7:122. doi: 10.1038/s41746-024-01115-7 (PMC11087561; doi:10.1038/s41746-024-01115-7)
Supplement: Supplementary file 1 — SUPPLEMENTAL MATERIAL [file 41746_2024_1115_MOESM1_ESM.docx]

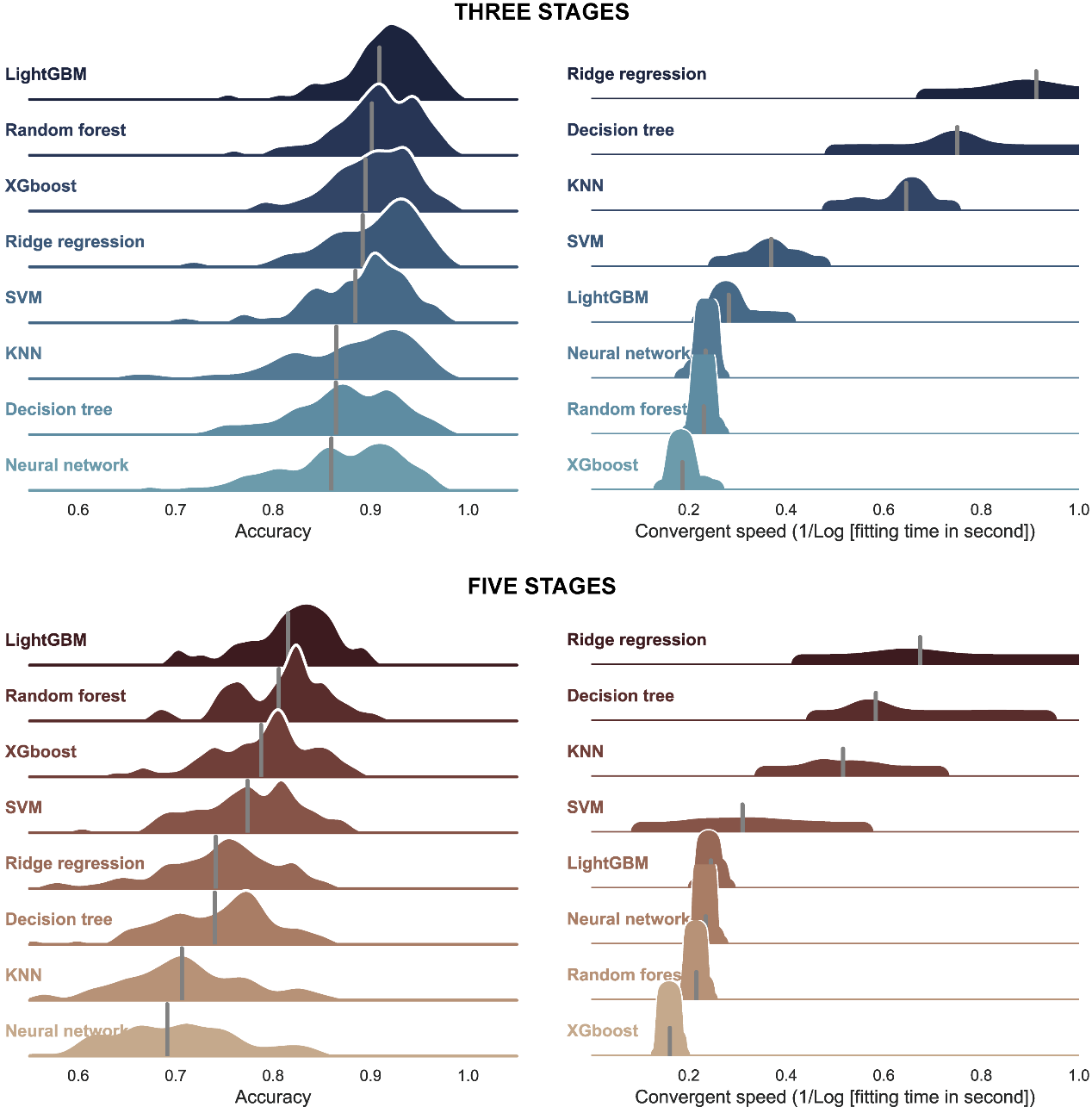


**Supplementary Figure 1** Evaluation of model performance in eight candidate classifiers. The one-side violin plot shows the probability density of the accuracy or convergent speed at different values. The vertical gray line represents the mean value of the accuracy or convergent speed.


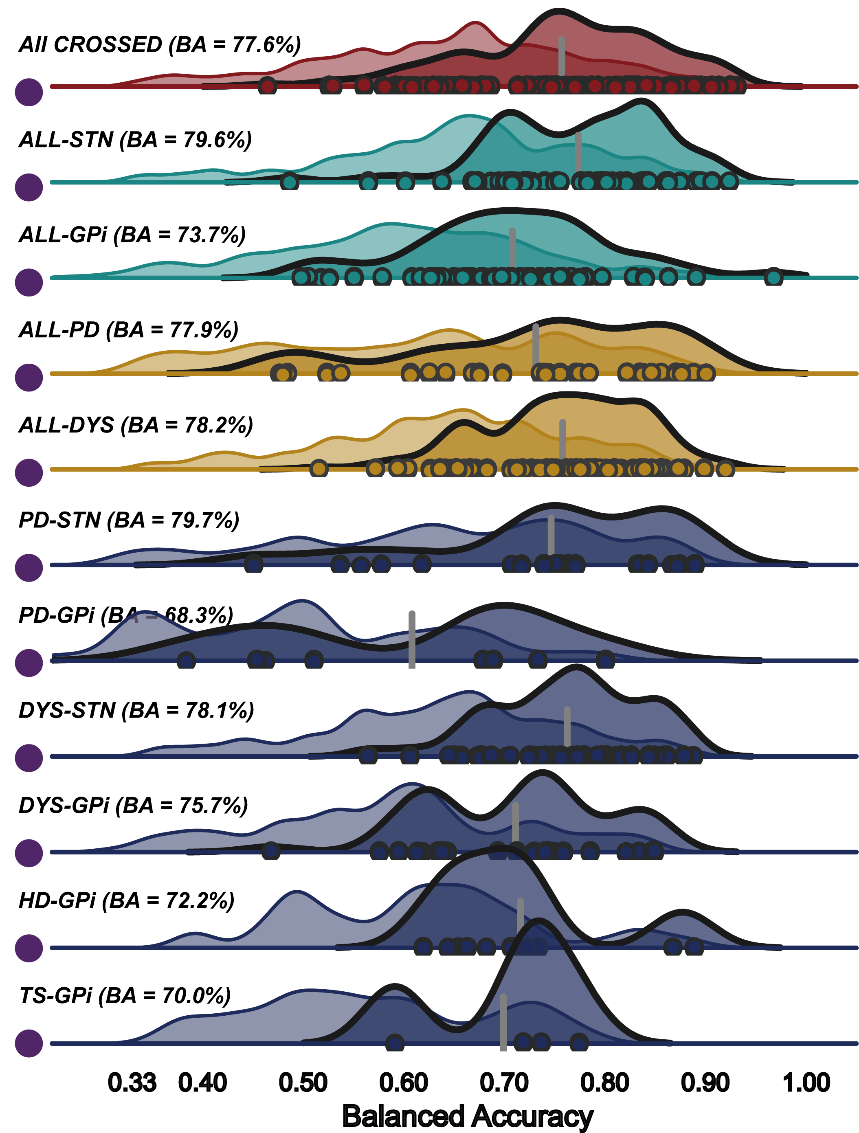


**Supplementary Figure 2** Cross-subject sleep decoding with basal ganglia signals for patients with movement disorders evaluated using balanced accuracies. The same convention as in Figure 3. BA, balanced accuracy.


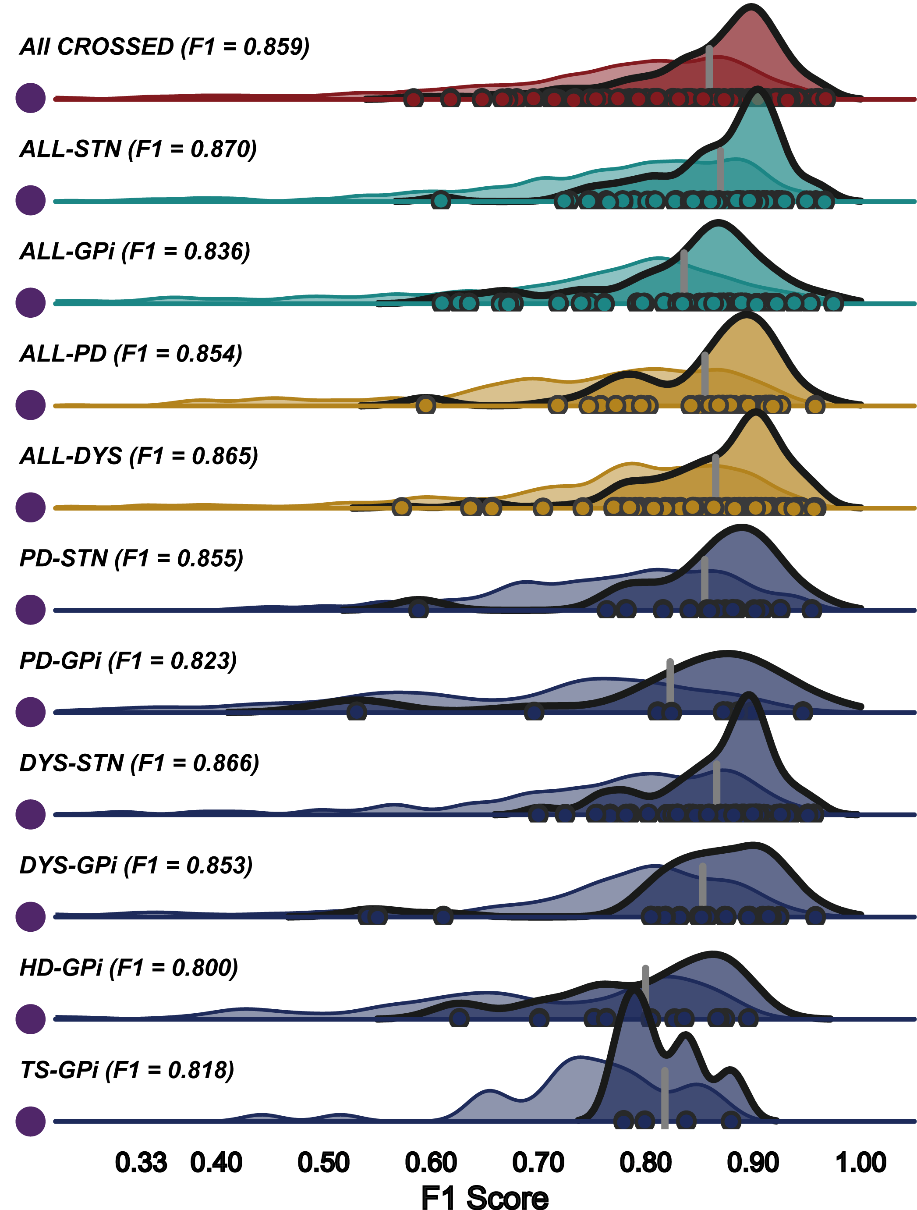


**Supplementary Figure 3** Cross-subject sleep decoding with basal ganglia signals for patients with movement disorders evaluated using F1 scores. The same convention as in Figure 3.


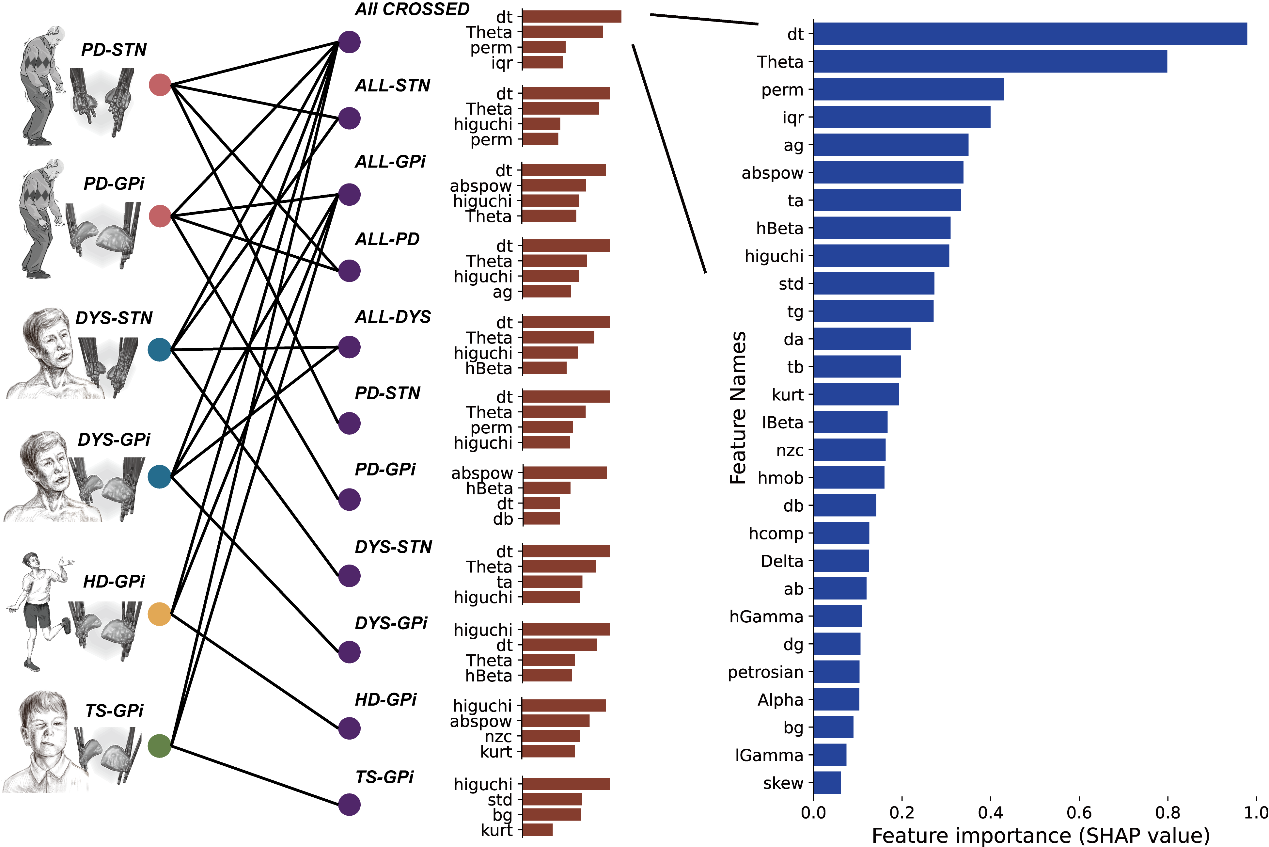


**Supplementary Figure 4** SHAP (SHapley Additive exPlanations) feature importance analysis for the cross-subject sleep decoding. The top four most contributed features in each decoding context are shown in red in the middle column. The importance of all features for the *ALL-CROSSED* decoding is shown in blue in the right column. dt, power ratio between delta and theta bands. Theta, theta band power. perm, permutation entropy. iqr, interquartile range. ag, power ratio between alpha and gamma bands. abspower, absolute power. ta, power ratio between theta and alpha bands. hBeta, high beta power. higuchi, Higuchi fractal dimension. std, standard deviation. tg, power ratio between theta and gamma bands. da, power ratio between delta and alpha bands. tb, power ratio between theta and beta bands. kurt, kurtosis. lBeta, low beta power. nzc. number of zero crossings. hmob, Hjorth mobility. db, power ratio between delta and beta bands. hcomp, Hjorth complexity. Delta, delta band power. ab, power ratio between alpha and beta bands. hGamma, high gamma band power. dg, power ratio between delta and gamma bands. petrosian, Petrosian fractal dimension. Alpha, alpha band power. bg, power ratio between beta and gamma bands. lGamma, low gamma band power. skew, skewness.


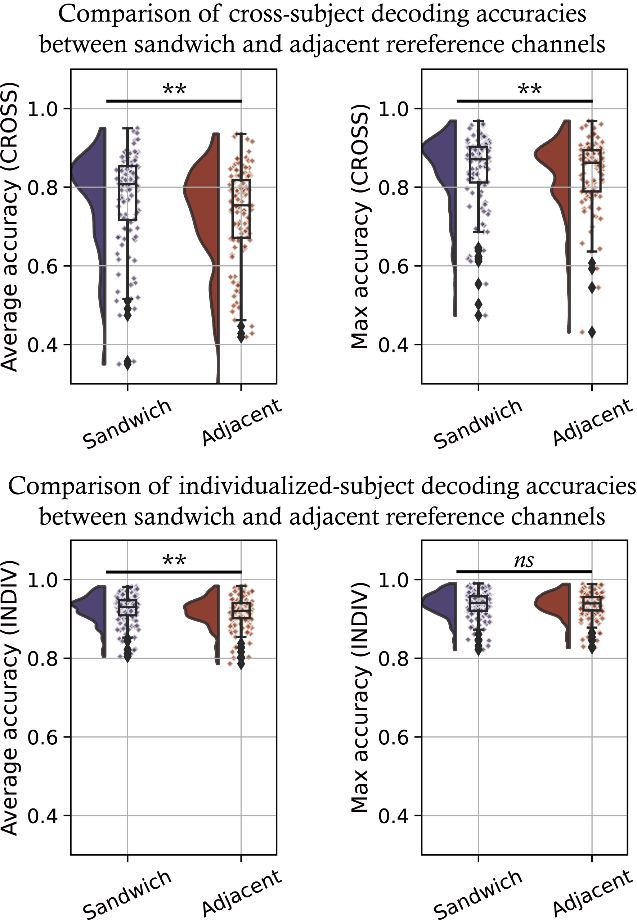


**Supplementary Figure 5** Comparison of decoding accuracies between sandwich and adjacent re-referenced basal ganglia channels. The top row shows the results of cross-subject decoding and the bottom row shows the results of individualized subject decoding. Figures in the left panel show the comparison of average accuracies (e.g., the average of 1-2, 2-3, 3-4 channels). Figures in the right panel show the comparison of max accuracies (e.g., the max of 1-2, 2-3, 3-4 channels). *P***=9.51×10^−12^ for the comparison of average cross-subject decoding accuracy between sandwich and adjacent re-referenced channels. *P***=8.89×10^−3^ for the comparison of max cross-subject decoding accuracy between sandwich and adjacent re-referenced channels. *P***=6.45×10^−15^ for the comparison of average individualized subject decoding accuracy between sandwich and adjacent re-referenced channels. *P*=0.317 for the comparison of max individualized subject decoding accuracy between sandwich and adjacent re-referenced channels. Wilcoxon signed-rank test.


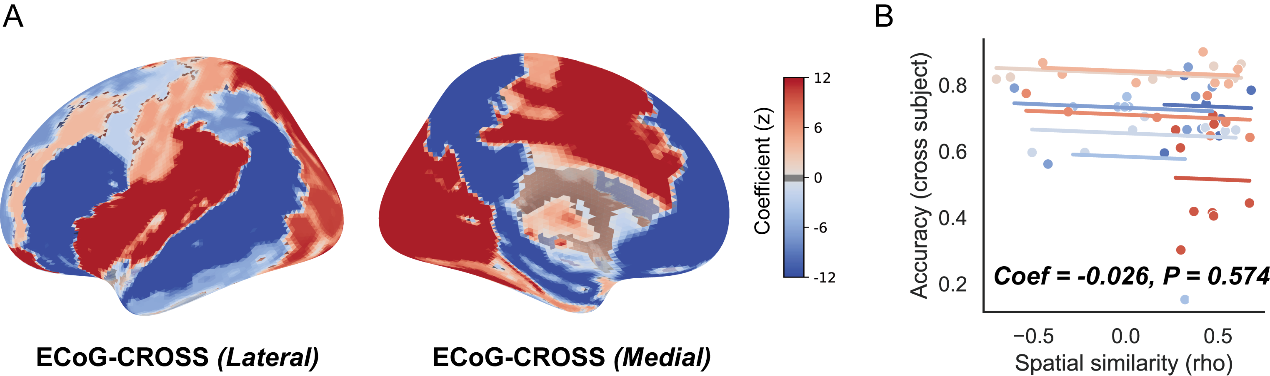


**Supplementary Figure 6** Network mapping of the decoding accuracies in ECoG channels. **A** shows the lateral and medial view of the optimal decoding map generated using ECoG data. **B** shows the repeated measurement regression plot between the spatial similarity to the optimal map and the decoding accuracies obtained in a leave-one-subject-out manner. The coefficient and p values were generated through the linear mixed effect model.


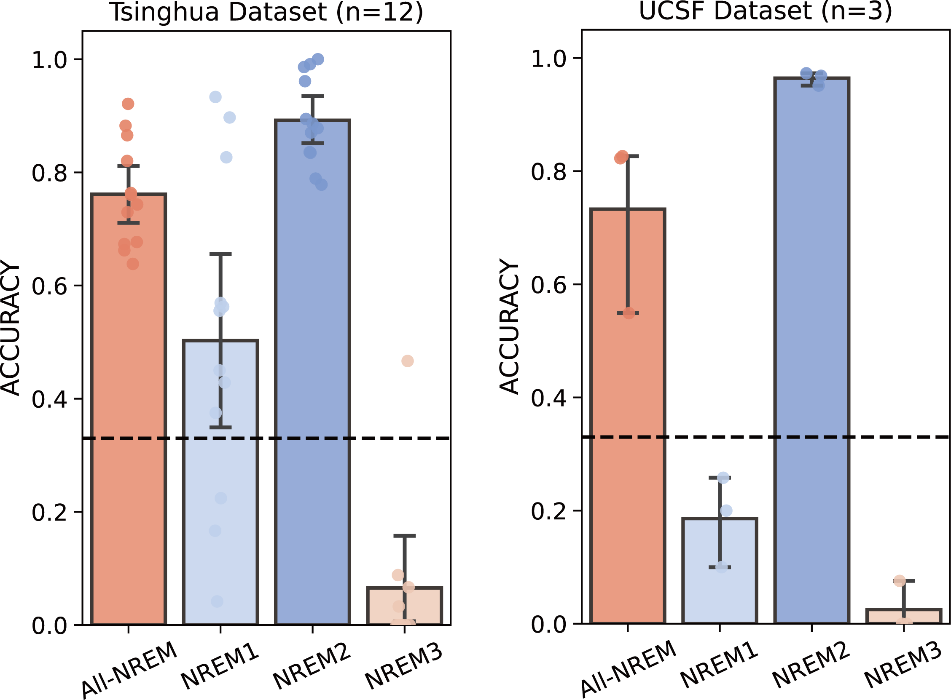


**Supplementary Figure 7** Performance of *BGOOSE* in classifying NREM stages in external datasets. For the Tsinghua dataset, the average accuracies of classifying all NREM, N1, N2, and N3 stages were 76.1±8.9%, 50.3±27.1%, 89.2±7.4%, and 6.5±13.7%, respectively. For the UCSF dataset, the average accuracies of classifying all NREM, N1, N2, and N3 stages were 73.3±12.9%, 18.6±6.5%, 96.4±0.9%, and 2.5±3.5%, respectively.


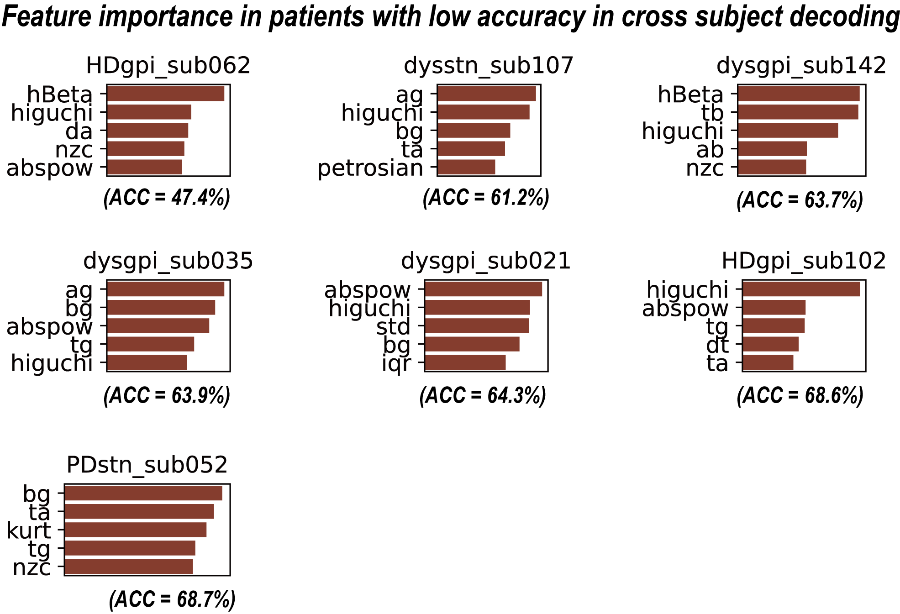


**Supplementary Figure 8** Feature importance in patients with lower-than-70% cross-subject decoding accuracies. See the caption of Supplementary Figure 4 for the full names of abbreviated feature names.

| Supplementary Table 1 The number of each sleep stage in all cross-subject decoding conditions^a^ | | | | | | | |
| --- | --- | --- | --- | --- | --- | --- | --- |
|  | All stages | Wake | NREM | REM | N1 | N2 | N3 |
| All-crossed | 71927 | 9836 | 50511 | 11580 | 2610 | 41167 | 6734 |
| All-STN | 45829 | 6034 | 32269 | 7526 | 1845 | 25977 | 4447 |
| All-GPi | 16059 | 2310 | 11449 | 2300 | 586 | 9224 | 1639 |
| All-PD | 40928 | 5593 | 28978 | 6357 | 1796 | 22983 | 4199 |
| All-DYS | 30999 | 4243 | 21533 | 5223 | 814 | 18184 | 2535 |
| PD-STN | 2682 | 215 | 1849 | 618 | 22 | 1502 | 325 |
| PD-GPi | 7357 | 1277 | 4944 | 1136 | 157 | 4464 | 323 |
| DYS-STN | 15669 | 2016 | 10896 | 2757 | 514 | 9199 | 1183 |
| DYS-GPi | 5291 | 735 | 3844 | 712 | 121 | 3019 | 704 |
| HD-GPi | 30160 | 4018 | 21373 | 4769 | 1331 | 16778 | 3264 |
| TS-GPi | 10768 | 1575 | 7605 | 1588 | 465 | 6205 | 935 |

^a^The number (n) was calculated as the sum of all 30-second segments in the corresponding sleep/wake stages. All stages represent the sum of the awake, NREM, and REM stages. NREM represents the sum of N1, N2, and N3 stages.

| Supplementary Table 2 The rate of mislabeling in all cross-subject decoding conditions^a^ | | | | | | | |
| --- | --- | --- | --- | --- | --- | --- | --- |
|  | All stages | Wake | REM | NREM | N1 | N2 | N3 |
| All-crossed | 17.7±10.2 | 6.0±6.1 | 4.6±5.4 | 7.2±9.0 | 1.2±2.3 | 3.8±4.6 | 2.2±4.2 |
| All-STN | 17.1±10.3 | 5.5±8.4 | 5.3±6.0 | 6.3±9.1 | 1.0±1.9 | 3.2±4.5 | 2.1±3.4 |
| All-GPi | 17.3±7.8 | 6.8±11.7 | 4.7±4.3 | 5.8±4.7 | 1.0±3.0 | 2.8±3.1 | 2.0±3.9 |
| All-PD | 16.0±7.6 | 5.6±7.8 | 4.7±4.8 | 5.7±5.7 | 1.0±2.2 | 2.6±3.6 | 2.1±4.3 |
| All-DYS | 21.0±11.9 | 7.2±8.8 | 5.7±5.8 | 8.1±10.3 | 0.9±2.0 | 4.5±5.4 | 2.8±5.0 |
| PD-STN | 19.7±4.8 | 7.4±6.4 | 8.1±8.0 | 4.2±5.2 | 0.2±0.6 | 1.3±4.3 | 2.7±8.2 |
| PD-GPi | 25.0±12.5 | 8.6±6.0 | 5.5±3.3 | 10.8±13.6 | 1.1±1.3 | 8.1±7.5 | 1.6±2.0 |
| DYS-STN | 20.2±14.8 | 5.4±8.3 | 7.3±6.0 | 7.5±8.2 | 1.2±2.1 | 4.4±4.1 | 2.0±2.5 |
| DYS-GPi | 20.4±12.7 | 8.0±8.7 | 7.8±6.5 | 4.6±3.8 | 0.6±2.3 | 1.3±2.5 | 2.7±6.6 |
| HD-GPi | 16.5±7.6 | 5.2±4.7 | 5.1±3.8 | 6.2±5.2 | 1.2±1.9 | 2.8±3.3 | 2.3±3.4 |
| TS-GPi | 16.9±7.6 | 6.4±7.7 | 4.4±2.8 | 6.1±5.4 | 1.1±2.9 | 2.8±3.2 | 2.2±5.0 |

^a^The rate of mislabeling was measured as the number of mislabeled 30-second segments per hour. For each substage, the mislabeling rate was calculated as the number of mislabeled segments in this substage divided by the total number of sleep segments of the night and then multiplied by 120. All stages represent the sum results of the awake, REM, and NREM sleep. NREM represents the sum results of N1, N2, and N3 sleep.

| Supplementary Table 3 Patient information for the external validation cohort | | | | | | |
| --- | --- | --- | --- | --- | --- | --- |
| Cohort-ID | Age | Gender | Diagnosis | Dx | DBS target | Epoch number |
| Tsinghua-01 | 40 | M | PD | 8 | STN | 194 |
| Tsinghua-02 | 53 | M | PD | 13 | STN | 328 |
| Tsinghua-03 | 67 | M | PD | 8 | STN | 430 |
| Tsinghua-04 | 51 | M | PD | 20 | STN | 475 |
| Tsinghua-05 | 65 | M | PD | 8 | STN | 487 |
| Tsinghua-06 | 60 | F | PD | 7 | STN | 502 |
| Tsinghua-07 | 46 | M | PD | 7 | STN | 469 |
| Tsinghua-08 | 61 | F | PD | 8 | STN | 339 |
| Tsinghua-09 | 47 | F | PD | 12 | STN | 441 |
| Tsinghua-10 | 56 | F | PD | 15 | STN | 195 |
| Tsinghua-11 | 61 | F | PD | 8 | STN | 527 |
| Tsinghua-12 | 51 | M | PD | 8 | STN | 483 |
| UCSF-01 | 58 | M | PD | 11 | STN | 208 |
| UCSF-02 | 40 | M | PD | 9 | STN | 337 |
| UCSF-03 | 48 | M | PD | 13 | GPi | 374 |

Epoch number represents the number of 30-second epochs used for model validation. Dx = Disease duration (years).

| Supplementary Table 4 Bayesian Optimization Hyperparameters | |
| --- | --- |
| **Hyperparameters** | **Ridge Regression** |
| ridgeclassifier__alpha | 0.001 to 1 |
| **Hyperparameters** | **Support Vector Machine** |
| svc__C | 0.001 to 1000 |
| svc__gamma | 0.0001 to 100 |
| svc__kernel | ['linear', 'poly', 'rbf', 'sigmoid'] |
| **Hyperparameters** | **K- Nearest Neighbor** |
| kneighborsclassifier__n_neighbors | 3 to 30 |
| kneighborsclassifier__weights | ['uniform', 'distance'] |
| kneighborsclassifier__metric | ['minkowski', 'euclidean', 'manhattan'] |
| **Hyperparameters** | **Decision Tree** |
| decisiontreeclassifier__max_depth | 1 to 100 |
| decisiontreeclassifier__min_samples_leaf | 1 to 4 |
| decisiontreeclassifier__min_samples_split | 2 to 10 |
| decisiontreeclassifier__criterion | ['gini', 'entropy'] |
| **Hyperparameters** | **Random Forest** |
| randomforestclassifier__n_estimators | 10 to 500 |
| randomforestclassifier__max_depth | 1 to 100 |
| randomforestclassifier__min_samples_leaf | 1 to 4 |
| randomforestclassifier__min_samples_split | 2 to 10 |
| **Hyperparameters** | **XGBoost** |
| xgbclassifier__n_estimators | 10 to 500 |
| xgbclassifier__max_depth | 1 to 100 |
| xgbclassifier__learning_rate | 0.01 to 1 |
| xgbclassifier__gamma | 1 to 10 |
| xgbclassifier__booster | ['gbtree'] |
| **Hyperparameters** | **LightGBM** |
| lgbmclassifier__n_estimators | 10 to 5000 |
| lgbmclassifier__max_depth | 1 to 100 |
| lgbmclassifier__num_leaves | 10 to 1000 |
| lgbmclassifier__feature_fraction | 0.1 to 1 |
| **Hyperparameters** | **Artificial Neural Network** |
| kerasclassifier__hidden_layers | [1, 2] |
| kerasclassifier__hidden_layer_dim | [16, 32, 64, 128] |
| kerasclassifier__optimizer__learning_rate | 0.0001 to 0.1 |
| kerasclassifier__activation | ['relu'] |
| kerasclassifier__loss | ['sparse_categorical_crossentropy'] |
| kerasclassifier__optimizer | ['adam'] |
