# Supplementary material for: Generalized sleep decoding with basal ganglia signals in multiple movement disorders
Source: NPJ Digit Med. 2024 May 10;7:122. doi: 10.1038/s41746-024-01115-7 (PMC11087561; doi:10.1038/s41746-024-01115-7)
Supplement: Supplementary file 2 — Reporting Summary [file 41746_2024_1115_MOESM2_ESM.pdf]

nature portfolio

Corresponding author(s): Jianguo Zhang

Last updated by author(s): Apr 1, 2024

Reporting Summary

Nature Portfolio wishes to improve the reproducibility of the work that we publish. This form provides structure for consistency and transparency in reporting. For further information on Nature Portfolio policies, see our Editorial Policies and the Editorial Policy Checklist.

Statistics

For all statistical analyses, confirm that the following items are present in the figure legend, table legend, main text, or Methods section.

n/a	Confirmed	
☐	☒	The exact sample size (*n*) for each experimental group/condition, given as a discrete number and unit of measurement
☐	☒	A statement on whether measurements were taken from distinct samples or whether the same sample was measured repeatedly
☐	☒	The statistical test(s) used AND whether they are one- or two-sided<br>*Only common tests should be described solely by name; describe more complex techniques in the Methods section.*
☐	☒	A description of all covariates tested
☐	☒	A description of any assumptions or corrections, such as tests of normality and adjustment for multiple comparisons
☐	☒	A full description of the statistical parameters including central tendency (e.g. means) or other basic estimates (e.g. regression coefficient) AND variation (e.g. standard deviation) or associated estimates of uncertainty (e.g. confidence intervals)
☐	☒	For null hypothesis testing, the test statistic (e.g. *F*, *t*, *r*) with confidence intervals, effect sizes, degrees of freedom and *P* value noted<br>*Give P values as exact values whenever suitable.*
☒	☐	For Bayesian analysis, information on the choice of priors and Markov chain Monte Carlo settings
☐	☒	For hierarchical and complex designs, identification of the appropriate level for tests and full reporting of outcomes
☐	☒	Estimates of effect sizes (e.g. Cohen's *d*, Pearson's *r*), indicating how they were calculated

Our web collection on statistics for biologists contains articles on many of the points above.

Software and code

Policy information about availability of computer code

Data collection	We used the JE-212 amplifier (Nihon Kohden, Tokyo, Japan) to record PSG and LFP data. For the out-of-cohort dataset, the Tsinghua dataset was recorded using the PINS perceptive DBS device. The UCSF dataset was recorded using the Medtronic sensing-enabled RC+S Summit devices.
Data analysis	We used Python 3.8/3.10 (packages including scikit-learn, [version 1.4.1], MNE-Python [verision 1.2.3], SciPy [version 1.7.3] and NumPy [version 1.20.2]) for the analysis of neurophysiological data.<br>We used MATLAB 2019b (LEAD-DBS [version 2.5.3]) to process all neuroimaging data.<br>All relevant codes employed in the study can be freely accessed at https://github.com/zixiao-yin/BGOOSE

For manuscripts utilizing custom algorithms or software that are central to the research but not yet described in published literature, software must be made available to editors and reviewers. We strongly encourage code deposition in a community repository (e.g. GitHub). See the Nature Portfolio guidelines for submitting code & software for further information.

Data

Policy information about availability of data

All manuscripts must include a data availability statement. This statement should provide the following information, where applicable:

- Accession codes, unique identifiers, or web links for publicly available datasets
- A description of any restrictions on data availability
- For clinical datasets or third party data, please ensure that the statement adheres to our policy

The original data are not yet openly available, as it is being used in ongoing projects. We welcome enquires for sharing this as part of a collaboration, please contact the corresponding authors.

Research involving human participants, their data, or biological material

Policy information about studies with human participants or human data. See also policy information about sex, gender (identity/presentation), and sexual orientation and race, ethnicity and racism.

Reporting on sex and gender	This study included 141 patients with movement disorder. Consent has been obtained for reporting individual-level but de-identified data. As a side analysis, We investigated how sex would influence the decoding accuracy of sleep stages. We found no significantly impact of sex on decoding accuracies, though.
Reporting on race, ethnicity, or other socially relevant groupings	Race, ethnicity, or other socially relevant variable were not used in our study because there is no sufficient evidence showing that these variables would have an impact on the decoding accuracy of sleep stages based on basal ganglia signals.
Population characteristics	A total of 141 patients were included, while 121 patients were analyzed in this manuscript. The average age of the 121 patients is 49.7 ± 16.9 years, and the average BMI is 22.4 ± 3.3.
Recruitment	A total of 141 patients with movement disorders scheduled to undergo DBS surgery at Beijing Tiantan Hospital were enrolled in the study after obtaining informed consent. Inclusion criteria comprised the following: 1) well-defined disease diagnosis; 2) ability to cooperate with whole-night PSG recordings; and 3) absence of structural lesions observed on MRI scans.<br>Patients were asked whether they are willing to participate in the study during lead externalization. Patients who agreed to participate may have higher degrees of sleep disturbances because those who have good sleep qualities may consider sleep monitoring unnecessary. The patients did not receive payment for their participation.
Ethics oversight	This study is in agreement with the Declaration of Helsinki, and is approved by the IRB of Beijing Tiantan Hospital (HX-A-2021006). All subjects provided written informed consent.

Note that full information on the approval of the study protocol must also be provided in the manuscript.

Field-specific reporting

Please select the one below that is the best fit for your research. If you are not sure, read the appropriate sections before making your selection.

☒ Life sciences ☐ Behavioural & social sciences ☐ Ecological, evolutionary & environmental sciences

For a reference copy of the document with all sections, see nature.com/documents/nr-reporting-summary-flat.pdf

Life sciences study design

All studies must disclose on these points even when the disclosure is negative.

Sample size	A total of 141 patients were included in this study. We employed no statistical models to predetermine sample size, but our sample sizes are at the same level as or larger than those reported in previous studies with similar research aims. For example, in 2018 JNNP paper [1], 10 PD patients were recruited for simultaneously PSG-LFP recordings during sleep; in 2018 TNSRE paper [2], 12 PD patients were recorded during sleep; and in 2022 NPJ Parkinsons Dis paper [3], 4 PD patients were recorded during sleep.<br>[1] Thompson J A, Tekriwal A, Felsen G, Ozturk M, Telkes I, Wu J, Ince N F, Abosch A. Sleep patterns in Parkinson's disease: direct recordings from the subthalamic nucleus[J]. Journal of Neurology, Neurosurgery & Psychiatry, 2018, 89(1): 95–104.<br>[2] Chen Y, Gong C, Hao H, Guo Y, Xu S, Zhang Y, Yin G, Cao X, Yang A, Meng F, Ye J, Liu H, Zhang J, Sui Y, Li L. Automatic Sleep Stage Classification Based on Subthalamic Local Field Potentials[J]. IEEE Trans Neural Syst Rehabil Eng, 2019, 27(2): 118–128.
Data exclusions	Data from 20 patients were excluded given the low count of NREM or REM sleep (< 5 minutes) for the decoding analysis.
Replication	All replications using the proposed BGOOSE model to decode sleep stages were successful.
Randomization	This is not relevant to our study as all included subjects are in the experimental group.
Blinding	Since this study is not related to interventions and therapeutic effects (i.e., we recorded brain signals from patients with movement disorders during sleep), blinding was not relevant to the study.

Reporting for specific materials, systems and methods

We require information from authors about some types of materials, experimental systems and methods used in many studies. Here, indicate whether each material, system or method listed is relevant to your study. If you are not sure if a list item applies to your research, read the appropriate section before selecting a response.

Materials & experimental systems

n/a	Involved in the study	
☒	☐	Antibodies
☒	☐	Eukaryotic cell lines
☒	☐	Palaeontology and archaeology
☒	☐	Animals and other organisms
☒	☐	Clinical data
☒	☐	Dual use research of concern
☒	☐	Plants

Methods

n/a	Involved in the study	
☒	☐	ChIP-seq
☒	☐	Flow cytometry
☐	☒	MRI-based neuroimaging

Plants

Seed stocks	Not applicable.
Novel plant genotypes	Not applicable.
Authentication	Not applicable.

Magnetic resonance imaging

Experimental design

Design type	Not applicable. We only used structure MRI (T1 sequence) for DBS electrode reconstruction
Design specifications	Not applicable.
Behavioral performance measures	Not applicable.

Acquisition

Imaging type(s)	Structure MRI
Field strength	3.o T
Sequence & imaging parameters	MRIs were acquired for all participants using a 3T Siemens Verio scanner with a T1-MPRAGE sequence [repetition time (TR) =2,300 ms, echo time (TE) =2.53 ms, flip angle =12°, slice thickness =1 mm, no gap, voxel size =1 mm × 1 mm × 1 mm]
Area of acquisition	Whole brain scan

Diffusion MRI ☐ Used ☒ Not used

Preprocessing

Preprocessing software	We used the advanced electrode localization pipeline (in Lead-DBS version 2.5.3) with default settings for electrode reconstruction and group analysis in Montreal Neurological Institute (MNI) space
Normalization	The pre-operative MRI scan (T1 sequence) was linearly co-registered with the postoperative anatomical CT and nonlinearly warped to the MNI template (ICBM 2009bNonlinear Asymmetric) using the Advanced Normalization Tools (ANTs)
Normalization template	ICBM 2009bNonlinear Asymmetric

Noise and artifact removal	Visual identification
Volume censoring	Not applicable

Statistical modeling & inference

Model type and settings	Not applicable
Effect(s) tested	Not applicable

Specify type of analysis: ☐ Whole brain ☐ ROI-based ☐ Both

Statistic type for inference (See Eklund et al. 2016)	Not applicable
Correction	Not applicable

Models & analysis

n/a	Involved in the study
☒	☐ Functional and/or effective connectivity
☒	☐ Graph analysis
☒	☐ Multivariate modeling or predictive analysis
